# Prehabilitation as a Biologically Active Intervention Is Associated with the Remodeling of the Pancreatic Tumor-Immune Microenvironment

**DOI:** 10.3390/ijms27020943

**Published:** 2026-01-18

**Authors:** Renee Stubbins, Boris Li, Matthew Vasquez, Blythe K. Gorman, Joseph Zambelas, Kelvin Allenson, Atiya Dhala, Wenjuan Dong, Hong Zhao, Stephen Wong

**Affiliations:** 1 Houston Methodist Neal Cancer Center, Houston, TX 77030, USA; 2 Department of Systems Medicine and Bioengineering, Houston Methodist Research Institute, Houston, TX 77030, USA; 3 Advanced Cellular and Tissue Microscopy Core, Houston Methodist Research Institute, Houston Methodist Neal Cancer Center, Houston, TX 77030, USA; 4 Department of Pathology and Genomic Medicine, Houston Methodist Hospital, Houston, TX 77030, USA; 5 Department of Pathology and Laboratory Medicine, Weill Cornell Medicine at Houston Methodist, Houston, TX 77030, USA; 6 Department of Pathology and Genomic Medicine, Texas A&M University Naresh K. Vashisht College of Medicine, Houston Campus, Houston, TX 77030, USA; 7 Neuroscience PhD Program, Weill Cornell Medicine at Houston Methodist, Houston TX 77030, USA; 8 Department of Surgery, Houston Methodist Hospital, Houston, TX 77030, USA; 9 Department of Surgery, J.C. Walter Jr. Transplant Center, Center for Critical Care, Houston Methodist Hospital, Houston, TX 77030, USA; 10 Department of Medicine, Weill Cornell Medicine at Houston Methodist, Houston, TX 77030, USA

**Keywords:** multimodal prehabilitation, pancreatic tumor microenvironment, spatial profiling

## Abstract

Pancreatic ductal adenocarcinoma (PDAC) is highly lethal, and many patients cannot undergo curative surgery due to frailty. Multimodal prehabilitation: combining exercise, nutrition, and psychological support improves functional readiness, but its biological impact on the PDAC tumor microenvironment (TME) is unclear. In this exploratory pilot study, we profiled resected PDAC tissues from prehabilitation-treated patients and matched controls using NanoString GeoMx Digital Spatial Profiling across immune, tumor, and stromal compartments (n = 4). Transcriptomic signatures were analyzed via differential expression, pathway enrichment, and MCP-counter deconvolution; protein-level validation used multiplex immunofluorescence (n = 8). Ligand–receptor modeling assessed cell–cell communication, and prognostic relevance was evaluated in TCGA-PDAC (n = 178). Prehabilitation was associated with increased NK-cell cytotoxicity, interferon response, and chemokine recruitment, as well as higher neutrophil signatures (*p* < 0.01) and reduced fibroblast signatures (*p* < 0.05). Tumor regions showed lower MAPK and PI3K/AKT activity, while stroma exhibited decreased TGF-β and Wnt signaling. Immunofluorescence confirmed neutrophil infiltration and reduced fibroblast density. TCGA analysis linked neutrophil-high/fibroblast-low profiles to longer survival (1044.6 vs. 458.7 days, *p* = 0.0052). These findings suggest prehabilitation may promote a more immune-active, less fibrotic TME in PDAC, resembling transcriptional states associated with improved survival. Prospective studies integrating biological and clinical endpoints are warranted.

## 1. Introduction

Pancreatic ductal adenocarcinoma (PDAC) remains one of the most lethal solid malignancies, with a five-year survival rate below 12% [1]. Surgical resection remains the only potentially curative therapy, yet fewer than 20% of patients present with resectable disease, and many who are technically eligible are unable to proceed to surgery due to frailty, sarcopenia, or reduced cardiopulmonary reserve [2,3]. These challenges have intensified interest in strategies that optimize patients’ functional capacity before treatment.

Prehabilitation, broadly defined as a structured program designed to enhance physiological and psychological resilience prior to treatment, has emerged as a promising supportive intervention in surgical oncology. Multimodal prehabilitation programs typically integrate supervised exercise training, tailored nutritional support, and psychological or stress-reduction components. Across colorectal, lung, and hepatobiliary cancers, prehabilitation has been associated with improved quality of life [4,5,6]. In patients with PDAC, recent prospective studies have demonstrated gains in exercise tolerance, lean body mass, and overall readiness for surgery, along with early evidence of improved postoperative recovery [3,7,8].

Despite these encouraging clinical findings, the biological effects of prehabilitation on the tumor microenvironment (TME) remains largely unknown. PDAC is characterized by a dense desmoplastic stroma, driven by cancer-associated fibroblasts, extracellular matrix remodeling, and immunosuppressive signaling [9,10,11]. These features impede effector immune cell infiltration, foster tumor progression, and limit therapeutic response. Whether a systemic intervention such as prehabilitation can influence the cellular and molecular composition of this complex TME is an important and unresolved question.

Preclinical studies suggest that host-level physiological states may shape tumor behavior. Exercise can enhance immune surveillance, promote intratumoral cytotoxic T-cell activity, and normalize tumor vasculature in murine models of several cancer types [12,13,14]. Nutritional and metabolic optimization can modulate systemic inflammation, while psychological interventions can attenuate stress-associated neuroendocrine pathways that impair immune function [15,16]. These observations raise the possibility that multimodal prehabilitation could exert biological effects within the tumor and stromal compartments.

In this exploratory pilot study, we investigate the potential impact of multimodal prehabilitation on the PDAC tumor microenvironment. Using NanoString GeoMx Digital Spatial Profiling, multiplex immunofluorescence, and computational modeling, we characterize immune, tumor, and stromal regions in PDAC resections from prehabilitation-treated patients and matched controls. We hypothesize that prehabilitation may be associated with features of a more immune-active and less fibrotic TME, providing early insights into its potential role as a biologically active adjunct to PDAC care.

## 2. Results

### 2.1. Immune Compartment Features Associated with Prehabilitation

To investigate whether multimodal prehabilitation is associated with alterations in the PDAC tumor microenvironment, we performed spatial transcriptomic profiling on formalin-fixed paraffin-embedded tumor tissues obtained at surgical resection. Tumors from patients who completed a standardized four-week prehabilitation program (n = 6) were compared with tumors from matched control patients who did not undergo prehabilitation (n = 6; Table 1). Representative tumor regions containing epithelial tumor cells, desmoplastic stroma, and infiltrating immune cells were identified by a gastrointestinal pathologist and analyzed using the NanoString GeoMx Digital Spatial Profiling platform with the Cancer Transcriptome Atlas panel. Within each tissue section, eight regions of interest (ROI) were selected and segmented into immune (CD45^+^), tumor (PanCK^+^), and stromal (CD45^−^/PanCK^−^) compartments, enabling compartment-specific transcriptomic analysis.

**Figure 1:**
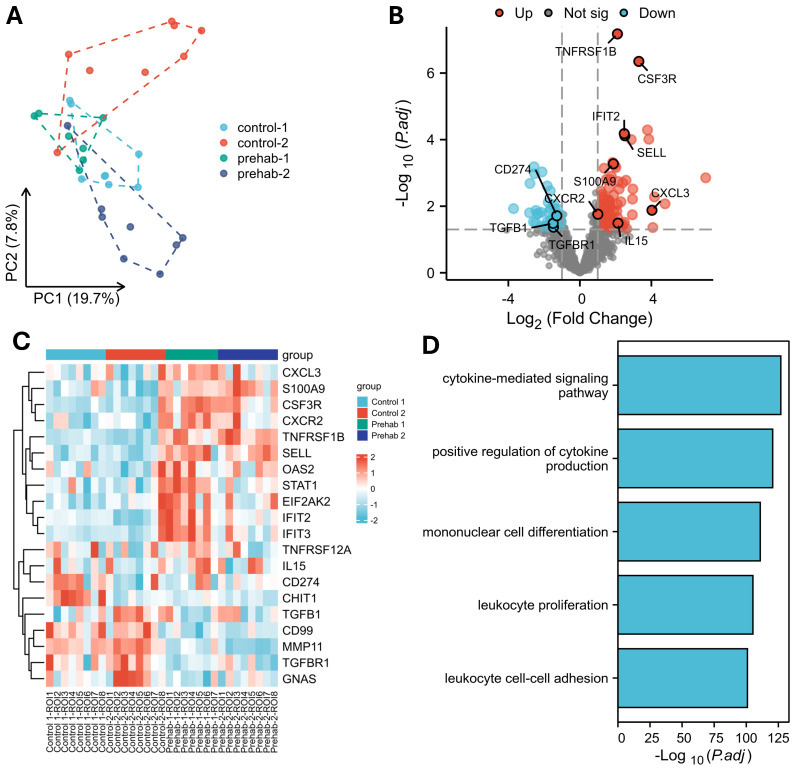
Prehabilitation remodels the immune compartment. (**A**) Spatial transcriptomic analysis showed strong variability among CD45^+^ immune ROIs within patients. Within each tissue section, eight ROIs were selected and segmented into immune (CD45^+^), tumor (PanCK^+^), and stromal (CD45^−^/PanCK^−^) compartments, enabling compartment-specific transcriptomic analysis. (**B**) Volcano plot comparing CD45^+^ regions from prehabilitation-treated and control tumors identified 770 differentially expressed genes (485 upregulated, 285 downregulated; adjusted *p* < 0.05, |log_2_FC| > 1). (**C**) Prehabilitation increased expression of CSF3R, CXCL3, CXCR2, SELL, TNFRSF1B, IL15, S100A9, and IFIT2, and down-regulated CD274 (PD-L1), TGFB1, and TGFBR1 in the immune ROIs. (**D**) Top pathways of the differentially expressed genes comparing CD45^+^ regions from prehabilitation-treated and control tumors.

Spatial transcriptomic profiling revealed substantial heterogeneity in CD45^+^ immune cell populations across multiple ROIs within each patient (Figure 1A). Despite this variability, consistent transcriptional differences were observed between prehabilitation-treated tumors and matched controls (Figure 1B). Differential expression analysis identified 770 significantly altered genes (adjusted *p* < 0.05, |log_2_FC| > 1), including 485 upregulated and 285 downregulated transcripts in the prehabilitation group.

Upregulated genes included *CSF3R*, *CXCL3*, *CXCR2*, *SELL*, and *S100A9*, suggesting enhanced neutrophil recruitment and responsiveness. Increased expression of *IL15* and *IFIT2* was also observed, consistent with heightened IL-15-associated support of NK and CD8^+^ T cells and an elevated type I interferon signaling environment (Figure 1C). Conversely, downregulated genes included *CD274* (PD-L1), *TGFB1*, and *TGFBR1*, which may reflect reduced transcriptional signatures of immune suppression within the tumor microenvironment.

Pathway-level analysis using Gene Ontology and Kyoto Encyclopedia of Genes and Genomes (GO/KEGG) combined with fold-change weighting demonstrated significant enrichment of cytokine-mediated signaling (Normalized Enrichment Score NES = 4.68, p.adjust = 2.14 × 10^−52^), positive regulation of cytokine production (NES = 4.24, p.adjust = 3.70 × 10^−44^), and lymphocyte activation pathways (NES = 4.30, p.adjust = 2.07 × 10^−32^) in prehabilitation-treated immune segments (Figure 1D, Supplementary Figure S1).

Together, these findings suggest that prehabilitation is associated with a more inflamed and recruitment-competent immune microenvironment in PDAC, characterized by enhanced chemokine signaling, elevated interferon activity, and transcriptional features supportive of lymphocyte activation.

### 2.2. Neutrophil-Associated Transcriptional Signatures in Prehabilitation-Treated Tumors

**Figure 2:**
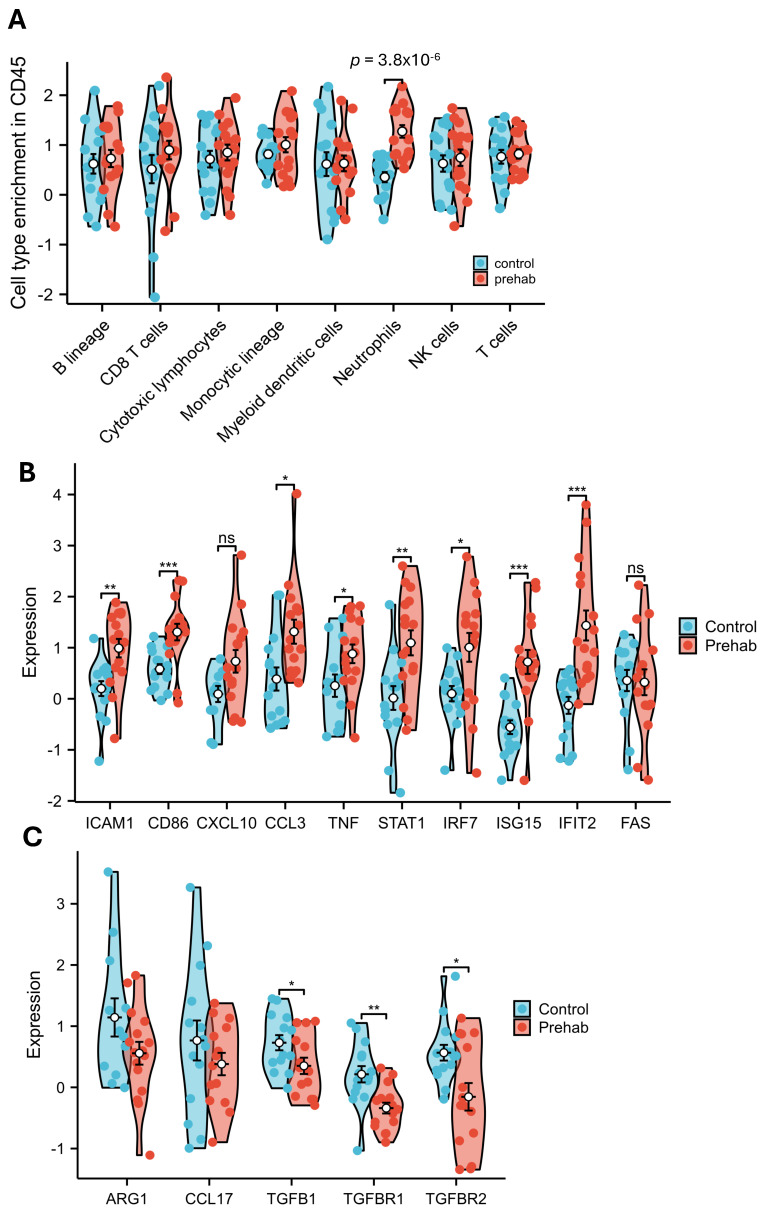
Neutrophil enrichment and N1 polarization with prehabilitation. (**A**) MCPcounter deconvolution analysis showed a significant increase in neutrophil abundance in prehabilitation-treated tumors compared with controls (*p* = 3.8 × 10^−6^, Welch *t*-test). (**B**) Differential gene expression analysis revealed upregulation of N1-associated genes including *ICAM1*, *CD86*, *CXCL10*, *CCL3*, *TNF*, and *FAS*, along with type I interferon–response genes *STAT1*, *IRF7*, *ISG15*, and *IFIT2* (* *p* < 0.05; ** *p* < 0.01; *** *p* < 0.001, ns: non-significant, Mann–Whitney U test). (**C**) TGFB1 and its receptors TGFBR1 and TGFBR2 were downregulated, indicating suppression of the TGF-β signaling pathway that drives the immunosuppressive N2 neutrophil phenotype (* *p* < 0.05; ** *p* < 0.01; Welch’s *t*-test).

MCP-counter analysis [17] was used to identify immune populations associated with prehabilitation. Neutrophil scores were significantly higher in prehabilitation-treated tumors compared with controls (mean 0.82 vs. 0.35, *p* = 3.8 × 10^−6^) (Figure 2A), indicating a consistent enrichment of neutrophil-associated transcripts.

Examination of neutrophil-related genes showed increased expression of markers commonly associated with pro-inflammatory or antitumor neutrophil activity, including *ICAM1*, *CD86*, *CXCL10*, *CCL3*, *TNF*, and *FAS* (Figure 2B). Upregulation of type I interferon–responsive genes (*STAT1*, *IRF7*, *ISG15*, *IFIT2*) further supported a transcriptional environment favorable to inflammatory activation. In contrast, *TGFB1* and its receptors (*TGFBR1*/*TGFBR2*) were downregulated (Figure 2C), consistent with reduced activity of pathways often associated with immunoregulatory or suppressive neutrophil states.

Taken together, these findings suggest that prehabilitation is associated with increased neutrophil representation in the PDAC tumor microenvironment, accompanied by transcriptional signatures consistent with a pro-inflammatory, interferon-associated, or immune-engagement transcriptional program as described in prior neutrophil polarization studies [18,19]. As this inference is based on bulk spatial transcriptomic signatures rather than direct functional assays, the results should be interpreted as indicative of a shift toward N1-associated transcriptional programs rather than definitive phenotypic classification.

### 2.3. Reduced Oncogenic Signaling Signatures in Tumor Compartments Associated with Prehabilitation

**Figure 3:**
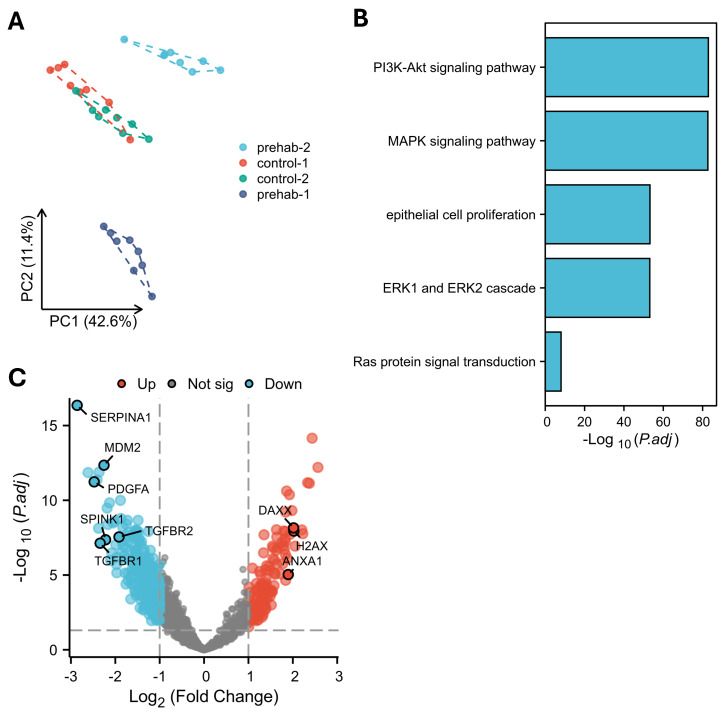
Prehabilitation suppresses oncogenic signaling in tumor compartments. (**A**) Principal component analysis showed inter-patient variability among PanCK^+^ tumor cell ROIs. (**B**) Top pathways of the differentially expressed genes comparing PanCK^+^ epithelial tumor regions from prehabilitation-treated and control tumors. (**C**) Volcano plot comparing PanCK^+^ epithelial tumor regions from prehabilitation-treated and control tumors showing transcript levels of key oncogenic drivers such as *SERPINA1*, *MDM2*, *PDGFA*, *TGFBR1*, *TGFBR2*, and *SPINK1* were reduced, while genes associated with DNA-damage response and apoptosis, including *H2AX*, *DAXX*, and *ANXA1*, were upregulated.

In the epithelial tumor (PanCK^+^) compartments, spatial transcriptomic profiling revealed broad differences in signaling pathway activity between prehabilitation-treated tumors and controls (Figure 3A). GO/KEGG combined with fold-change pathway enrichment analysis showed significantly lower activity of several oncogenic pathways in prehabilitation samples, including MAPK (NES = −4.17, p.adjust = 2.97 × 10^−16^), PI3K/AKT (NES = −4.91, p.adjust = 1.31 × 10^−18^), and Ras signaling (NES = −3.93, p.adjust = 5.84 × 10^−16^) (Figure 3B, Supplementary Figure S2).

These pathway-level changes were accompanied by reduced expression of transcripts frequently associated with tumor-promoting programs, such as *SERPINA1*, *MDM2*, *PDGFA*, *TGFBR1/2*, and *SPINK1* (Figure 3C). Conversely, genes linked to DNA-damage responses and apoptotic pathways (*H2AX*, *DAXX*, *ANXA1*) demonstrated relatively higher expression in prehabilitation-treated tumor regions.

Together, these transcriptional profiles suggest that prehabilitation is associated with reduced activity of proliferative and oncogenic signaling programs in epithelial tumor cells. While these findings do not establish causality, they highlight potentially meaningful alterations in tumor-intrinsic signaling that warrant further investigation in larger, mechanistically focused studies.

### 2.4. Stromal Features and Reduced Immunosuppressive Signaling Associated with Prehabilitation

**Figure 4:**
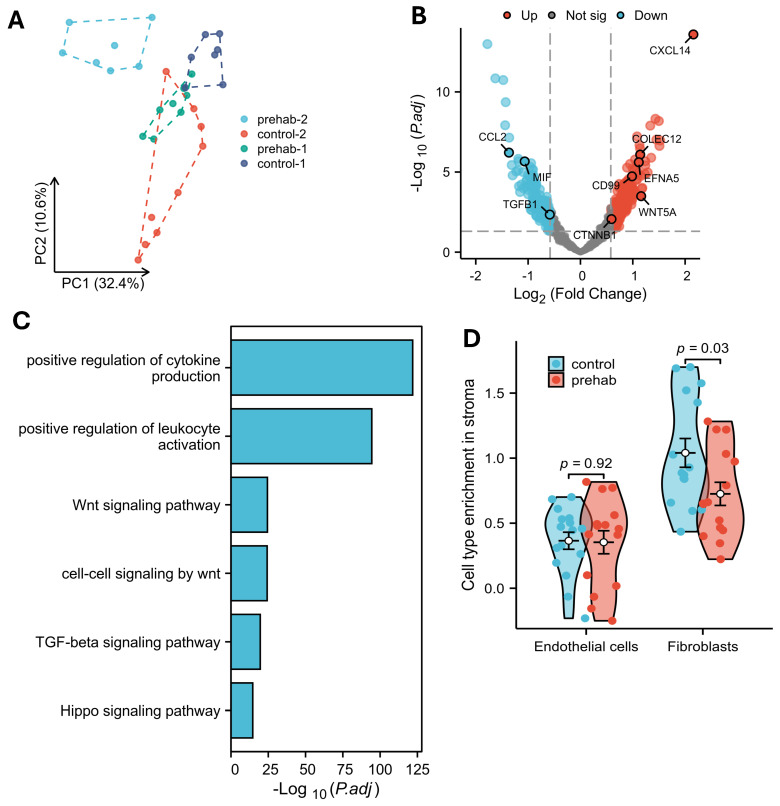
Stromal remodeling and reduced immunosuppression with prehabilitation. (**A**) Principal component analysis showed inter-patient variability among stromal (CD45^−^/PanCK^−^) regions. (**B**) Volcano plot comparing stromal (CD45^−^/PanCK^−^) regions from prehabilitation-treated and control tumors. (**C**) Top pathways of the differentially expressed genes comparing PanCK^−^/CD45^−^ stromal regions from prehabilitation-treated and control tumors. (**D**) MCP-counter analysis showed fewer fibroblasts in prehabilitation-treated tissues compared with controls (*p* = 3.8 × 10^−6^, Welch t’ test).

Spatial profiling of stromal (CD45^−^/PanCK^−^/Syto13^+^) cellular compartments revealed transcriptional differences between prehabilitation-treated and control tumors (Figure 4A). Several transcripts associated with immunosuppressive or protumorigenic stromal programs, including *TGFB1*, *MIF*, and *CCL2*, were reduced in prehabilitation samples (Figure 4B). Correspondingly, gene set enrichment analysis indicated decreased activity of the TGF-β signaling pathway (NES = −3.02, p.adjust = 2.52 × 10^−11^) (Figure 4C, Supplementary Figure S3).

Transcripts linked to Wnt and Hippo signaling—such as *CTNNB1* and *WNT5A*—were also downregulated (Figure 4B,C). These pathways have been implicated in fibroblast–tumor communication and exclusion of effector immune cells from the PDAC microenvironment [20]. In contrast, stromal regions from prehabilitation-treated tumors demonstrated increased expression of genes such as *CXCL14*, *COLEC12*, *EFNA5*, and *CD99*, which have been associated with enhanced stromal–immune interactions [21].

GO/KEGG enrichment analysis supported these observations, showing increased activity of pathways related to cytokine production (NES = 2.34, p.adjust = 2.34 × 10^−12^), leukocyte activation (NES = 1.09, p.adjust = 1.39 × 10^−37^), and regulation of immune effector processes (NES = 1.34, p.adjust = 8.51 × 10^−40^) (Figure 4C, Supplementary Figure S3). In line with these transcriptomic signatures, MCP-counter analysis demonstrated significantly lower fibroblast scores in prehabilitation-treated tumors compared with controls (mean 0.82 vs. 0.35, *p* = 3.8 × 10^−6^) (Figure 4D).

Together, these findings suggest that prehabilitation is associated with reduced expression of stromal immunosuppressive programs and increased transcriptional features linked to stromal–immune communication. These results should be interpreted as indicative of stromal remodeling at the gene expression level, recognizing that functional validation in larger cohorts will be required to confirm the biological impact of these observations.

### 2.5. Multiplex Immunofluorescence Validates Stromal and Immune Remodeling in Prehabilitation Tumors

**Figure 5:**
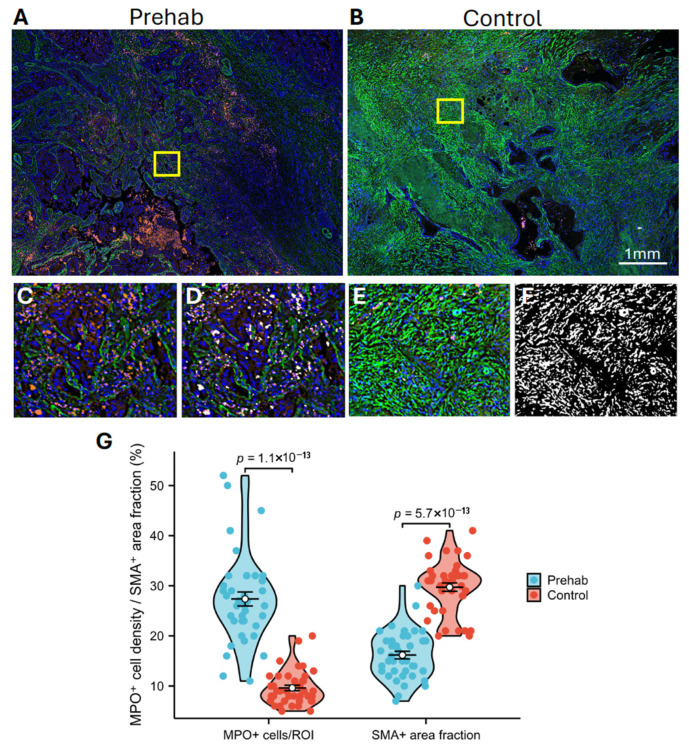
Multiplex immunofluorescence showing stromal and immune changes with prehabilitation. (**A**,**B**) Representative dual-color IF images of prehabilitation-treated and control PDAC tissue sections. (**C**,**E**) Zoomed-in views of the yellow boxed regions. (**D**,**F**) Corresponding binary masks highlighting MPO^+^ cells (white in (**D**)) and SMA^+^ fibroblast areas (white in (**F**)). Red: MPO^+^ neutrophils; green: SMA^+^ fibroblast areas; blue: nuclei. (**G**) Quantification of MPO^+^ cell density and SMA^+^ area fraction. Data represent 4 tissue slides per group, with 10 ROIs per slide (each ROI, 500 μm diameter). *p*-values were calculated using the Wilcoxon rank sum test.

To independently assess the transcriptomic findings at the protein level, dual-plex multiplex immunofluorescence was performed in a separate validation cohort (n = 8) (Figure 5A–F). Consistent with the spatial transcriptomic signatures, prehabilitation-treated tumors showed significantly greater infiltration of MPO^+^ neutrophils compared with controls (23.6 ± 4.1 cells/μm^2^ vs. 8.7 ± 2.9 cells/μm^2^, *p* = 1.1 × 10^−13^). In parallel, the proportion of α-SMA^+^ fibroblast area within stromal regions was markedly reduced in prehabilitation samples (15.3 ± 3.2 percent vs. 32.5 ± 5.7 percent, *p* = 5.7 × 10^−13^) (Figure 5G).

These protein-level differences align with the transcriptomic observations of increased neutrophil-associated signatures and decreased fibroblast representation, supporting the association between prehabilitation and coordinated stromal and immune remodeling in PDAC tumors.

### 2.6. Prehabilitation Is Associated with Increased Immune Intake and Antigen-Presentation Signaling

**Figure 6:**
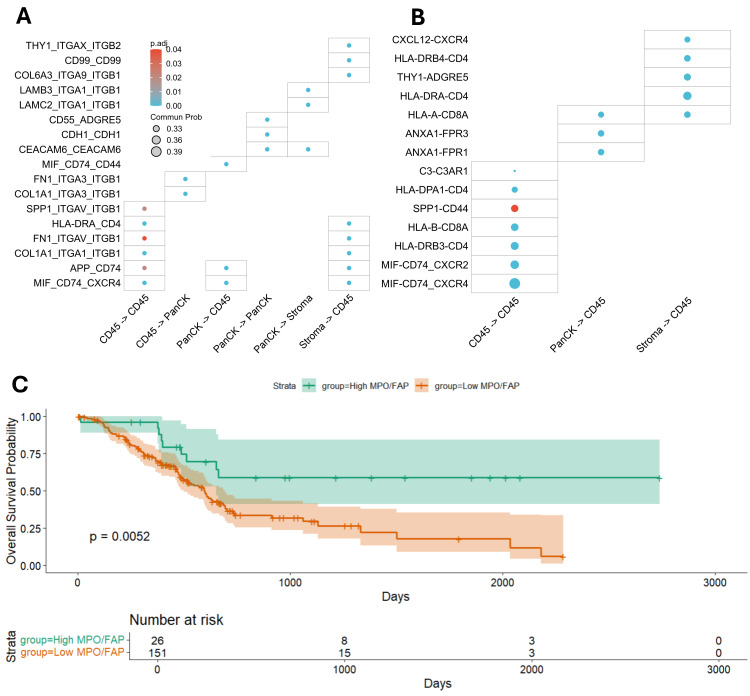
CellChat analysis of ligand–receptor communication and prognostic relevance of the Prehab-TME phenotype. (**A**) CellChat bubble plot of control tumors showing that signaling among PanCK^+^, stromal, and CD45^+^ compartments was dominated by extracellular matrix–integrin pathways, indicating limited immune engagement. (**B**) In prehabilitation-treated tumors, CellChat analysis revealed increased Stroma → CD45 and CD45 → CD45 interactions, reflecting enhanced immune communication. Bubble size represents communication probability, and color indicates adjusted *p*-values from CellChat permutation testing. (**C**) Kaplan–Meier analysis of overall survival in TCGA–PDAC patients stratified by the Prehabilitation-TME index phenotype. The Index High group (Neutrophil-high/Fibroblast-low) showed a trend toward longer overall survival compared with the Index Low group (Neutrophil-low/Fibroblast-high).

Ligand–receptor modeling was performed to assess compartment-level communication among tumor (PanCK^+^), stromal, and immune (CD45^+^) regions. In control tumors, inter-compartment signaling was primarily dominated by extracellular matrix and integrin-mediated interactions (Figure 6A). PanCK → CD45 signaling consisted largely of matrix-associated contacts such as *FN1/COL1A1–integrin* pairs, along with *APP → CD74*. Stromal → CD45 communication in control tumors was largely characterized by similar extracellular matrix and adhesion-associated interactions (for example, *FN1/COL1A1–integrin* pairs), with fewer immune-relevant edges including HLA-DRA → CD4, APP → CD74, and the MIF → CD74/CXCR4 axis. In contrast, prehabilitation-treated tumors exhibited broader and more diverse signaling toward CD45^+^ immune cells (Figure 6B). Stromal → CD45 communication featured increased chemotactic and immune-engagement signals, including *CXCL12 → CXCR4* and *HLA-DRA → CD4/CD8*, consistent with enhanced transcriptional signatures related to T-cell recruitment. PanCK → CD45 signaling gained interactions such as *HLA-A → CD8A* and *ANXA1 → FPR1/FPR3*, suggesting increased MHC class I–associated engagement of CD8^+^ T cells and augmented chemotactic signaling to myeloid cells.

Within the CD45 → CD45 network, antigen-presentation–associated interactions expanded, including *HLA-A/B → CD8A* and *HLA-DRA/DRB3/DRB4/DPA1 → CD4*. Adhesion and trafficking-related edges, such as *SPP1 → CD44*, were also strengthened. Although the immunoregulatory *MIF → CD74/CXCR4/CXCR2* axis persisted, it coexisted with a broader set of pro-inflammatory and immune-recruiting interactions.

Overall, these ligand–receptor patterns suggest that prehabilitation is associated with increased leukocyte-directed signaling, enhanced T-cell engagement, and features consistent with a more communication-active immune microenvironment.

### 2.7. Prognostic Validation in TCGA

To explore the potential clinical relevance of the stromal and immune features observed in prehabilitation-treated tumors, we evaluated a “prehabilitation-like TME index” in TCGA–PDAC, defined by the ratio of neutrophil-associated (*MPO*) to fibroblast-associated (*FAP*) expression, with the high-index group defined by the upper tail of the distribution rather than a median split. MPO and FAP were selected as representative markers of neutrophil-associated and activated fibroblast–associated expression, respectively, with the explicit acknowledgment that neither marker is cell-type exclusive in bulk RNA-seq data. This index approach was used to capture tumors with a pronounced immune-dominant and fibroblast-low transcriptional profile, which was relatively uncommon in the cohort (n = 26 vs. n = 151). The threshold was defined without outcome-based optimization, and the analysis was intended as exploratory and hypothesis-generating rather than prognostic validation. Patients with higher index values exhibited an improved overall survival compared with those in the lower index group (log-rank *p* = 0.0052) (Figure 6C). These analyses support the hypothesis that a tumor microenvironment characterized by higher neutrophil-associated signatures and lower fibroblast-associated signatures may be associated with more favorable outcomes.

## 3. Discussion

This exploratory study provides initial evidence that multimodal prehabilitation may be associated with biologically meaningful remodeling of the pancreatic ductal adenocarcinoma tumor microenvironment at both the transcriptomic and protein levels. Through spatial profiling, ligand–receptor modeling, and multiplex immunofluorescence validation, we identified convergent features consistent with increased immune activation, enhanced neutrophil-associated signaling, reduced fibroblast representation, and suppression of key oncogenic and stromal pathways. Collectively, these findings support our central hypothesis that prehabilitation, beyond improving physiological readiness for surgery, may also be linked to molecular and cellular alterations within the PDAC microenvironment.

A key novelty of this study is the spatially resolved characterization of the tumor, stromal and immune compartments following prehabilitation, an area for which molecular data have been largely absent. Prior studies of prehabilitation in PDAC have focused primarily on functional outcomes such as postoperative morbidity, length of stay, and physical performance, with limited investigation of tumor-intrinsic or microenvironmental effects [6,22]. By interrogating compartment-specific signaling, our data suggest that prehabilitation is associated with a shift toward a more immune-communicative tumor microenvironment, characterized by increased immune-directed signaling and reduced dominance of fibroblast-associated programs.

The enrichment of neutrophil-associated proinflammatory signatures observed in prehabilitated tumors is particularly notable. Exercise and physical conditioning are known to influence myelopoiesis, immune cell trafficking, and neutrophil activation states in both cancer and non-cancer settings [23,24]. In PDAC, neutrophils have historically been associated with immunosuppression and poor prognosis [25,26]; however, increasing evidence supports functional heterogeneity among tumor-associated neutrophils, with subsets capable of promoting antigen presentation, T-cell recruitment, and antitumor immunity [27,28]. The spatial enrichment of neutrophil-related transcriptional programs in conjunction with increased antigen-presentation and immune-engagement signaling observed here is therefore consistent with a shift toward a more immune-active neutrophil phenotype rather than a purely suppressive role.

Concurrently, we observed a reduction in fibroblast abundance and suppression of stromal signaling pathways, including TGF-β, Wnt, and Hippo signaling. Cancer-associated fibroblasts are a defining feature of PDAC and are central drivers of immune exclusion, extracellular matrix deposition, and resistance to both chemotherapy and immunotherapy [29,30]. Stromal-derived TGF-β signaling in particular has been implicated in immune suppression and poor response to immune checkpoint blockade [31]. The observed attenuation of fibroblast representation and stromal pathway activity in prehabilitated tumors suggests a potential mechanism by which prehabilitation could alleviate stromal-mediated immunosuppression and permit greater immune engagement.

Tumor-intrinsic signaling pathways were also altered. The downregulation of MAPK and PI3K/AKT signaling within tumor regions aligns with reduced proliferative and survival signaling, pathways that are frequently activated in PDAC and associated with aggressive disease biology and therapeutic resistance [32,33]. While causality cannot be inferred from this pilot cohort, these findings raise the possibility that systemic interventions such as prehabilitation may indirectly influence tumor cell signaling through remodeling of the surrounding microenvironment.

The potential clinical relevance of these microenvironmental states is supported by our TCGA-based analysis, in which a higher neutrophil-to-fibroblast index demonstrated a trend toward improved survival. Although limited by the small sample size and lack of clinical outcome data within our cohort, it is directionally consistent with prior work highlighting the prognostic importance of immune–stromal balance in PDAC [34,35] and supports the translational plausibility of the microenvironmental features identified in this study.

Together, these findings provide a framework for incorporating biological endpoints into future interventional trials of prehabilitation. Prospective randomized controlled trials could integrate paired pre- and post-intervention tissue sampling, longitudinal blood-based immune profiling, and quantitative stromal analyses to determine whether microenvironmental remodeling mediates improvements in postoperative recovery or oncologic outcomes. Circulating biomarkers, such as cytokine signatures, neutrophil activation markers, or stromal-derived factors, could serve as minimally invasive readouts of biological response [36]. Integrating these endpoints with standardized prehabilitation protocols would allow a more rigorous assessment of causality and help define which components of prehabilitation exert the most pronounced biological effects.

This study has limitations, including a small sample size, absence of long-term clinical outcome data, and inference of neutrophil states from transcriptomic rather than single-cell or functional analysis. In addition, while spatial profiling offers valuable compartment-level resolution, it does not capture temporal dynamics of immune–stromal interactions. Larger studies incorporating single-cell and spatial multi-omic approaches, expanded immunophenotyping, and mechanistic validation will be required to confirm and extend these preliminary observations.

Despite these limitations, our findings highlight the potential for prehabilitation to influence the PDAC microenvironment in ways that could extend beyond physical conditioning. Embedding biological endpoints into future trials may help determine whether prehabilitation can be leveraged not only to improve surgical readiness but also to modulate tumor biology and potentially enhance oncologic outcomes.

## 4. Materials and Methods

### 4.1. Patient Samples and Study Design

FFPE tumor tissues were obtained from patients undergoing surgical resection for PDAC at Houston Methodist Hospital. Prehabilitation participants (n = 6) completed a standardized four-week multimodal program. Six patients matched for age, sex, and tumor stage who did not undergo prehabilitation served as controls (Table 1). All procedures were approved by the Institutional Review Board, and written informed consent was obtained from all participants.

### 4.2. Prehabilitation Intervention

Patients in the prehabilitation group completed a standardized four-week multimodal program prior to surgical resection. The program consisted of structured exercise training, individualized nutritional optimization, and psychological support, consistent with published prehabilitation frameworks [3,37].

### 4.3. Exercise Regimen

Participants engaged in supervised sessions three times per week, delivered by an exercise physiologist. Each session included:

Aerobic training: 20–30 min of moderate-intensity activity (target 60–70 percent of age-adjusted maximal heart rate), performed on a treadmill, stationary cycle, or elliptical trainer based on patient preference and mobility.

Resistance training: 20 min of strength exercises targeting major muscle groups (1–2 sets of 10–15 repetitions at light-to-moderate intensity using bands or free weights).

Functional mobility: Low-impact balance and flexibility exercises (5–10 min) designed to enhance stability and gait.

Patients were encouraged to perform an additional home-based aerobic session once weekly; adherence was monitored through weekly check-ins.

### 4.4. Nutritional Optimization

All participants met with a registered dietitian at baseline and weekly thereafter. The nutrition plan emphasized: improving caloric intake to meet individualized energy requirements, ensuring adequate protein intake (typically 1.2–1.5 g/kg/day depending on baseline nutritional status), use of oral nutritional supplements when dietary intake was insufficient. Dietary adherence was monitored using brief weekly dietary recalls and counseling sessions.

### 4.5. Psychological and Behavioral Support

Patients received one structured weekly session with a trained counselor focusing on: stress-reduction strategies (guided breathing, relaxation techniques), coping skills for managing diagnosis-related anxiety, motivational interviewing to support adherence to exercise and nutrition components. Patients were also provided optional written and digital resources for at-home practice. Overall adherence to the prehabilitation program was assessed through weekly multidisciplinary review meetings, and participation across exercise, nutrition, and psychological components was documented.

### 4.6. Histopathologic Review and Spatial Transcriptomics

Hematoxylin- and eosin-stained slides from all cases were reviewed by a gastrointestinal pathologist to identify representative tumor regions. Areas containing epithelial tumor cells, desmoplastic stroma, and infiltrating immune cells were selected for spatial profiling. FFPE sections (5 μm) were stained with anti-CD45, anti-PanCK, and Syto-13 to enable automated segmentation of immune (CD45^+^), tumor (PanCK^+^), and stromal (CD45^−^/PanCK^−^) compartments.

**Figure 7:**
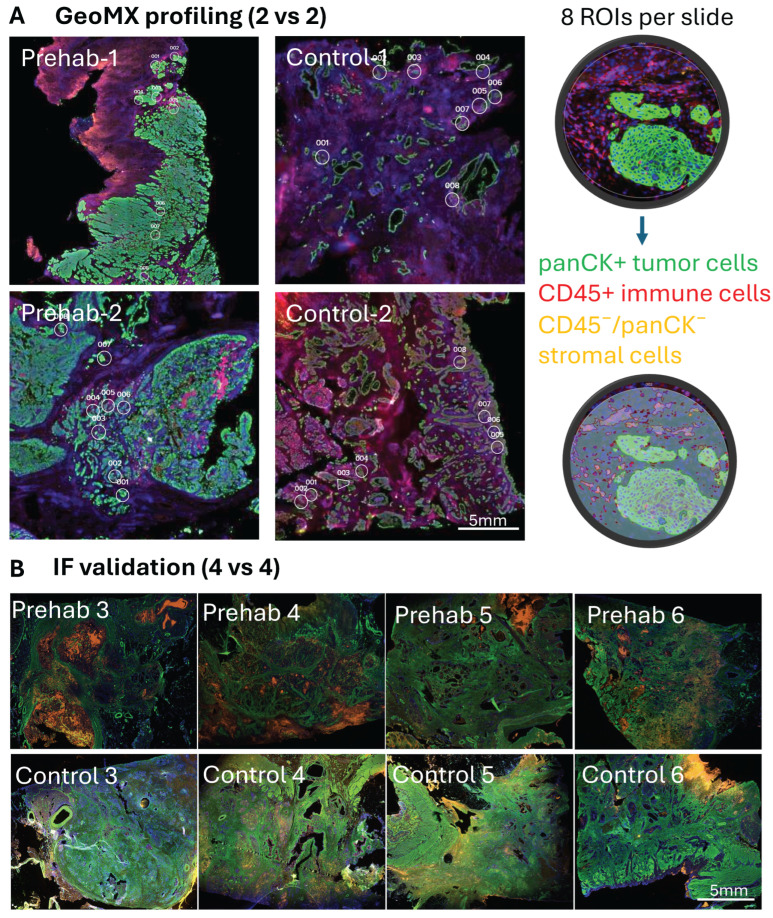
Spatial profiling and histologic validation of the tumor microenvironment. (**A**) Spatial transcriptomic analysis was performed using the NanoString GeoMx Digital Spatial Profiling (DSP) platform with a cancer transcriptome panel of 8600 probes. For each FFPE tissue section, eight circular regions of interest (ROIs; 300 μm diameter) were selected to capture representative tumor areas. Within each ROI, three biological compartments—immune (CD45^+^), tumor (PanCK^+^), and stromal (CD45^−^/PanCK^−^)—were segmented and profiled separately to generate spatially resolved transcriptomic data across the tumor microenvironment. (**B**) Dual-color multiplex immunofluorescence (mIF, 2-plex) was performed on single FFPE sections from the validation cohort (n = 8) to quantify neutrophils (MPO^+^) and myofibroblasts (α-SMA^+^). Fluorescent signals were analyzed to assess spatial abundance and compartment-specific distribution of these cell populations within the tumor microenvironment.

Spatial transcriptomic profiling was performed using the NanoString GeoMx Digital Spatial Profiling (DSP) platform with the Cancer Transcriptome Atlas panel (8600 probes) (Brucker Spatial Biology, Seattle WA, USA). For each tissue section, eight circular regions of interest (ROIs; 300 μm diameter) were selected. Within each ROI, three biological segments—immune, tumor, and stromal—were independently profiled (Figure 7A).

Raw counts were normalized to housekeeping genes and log_2_-transformed prior to analysis. Differential expression was performed at the ROI level using the Limma pipeline (version 3.x) [38], which is well suited for high-dimensional transcriptomic data with small sample sizes. Patient ID was included as a blocking factor, and duplicateCorrelation was applied to account for non-independence of multiple ROIs derived from the same tumor. Empirical Bayes–moderated t-statistics were used to stabilize variance estimates, and *p* values were adjusted using the Benjamini–Hochberg false discovery rate (FDR). Gene Ontology and Kyoto Encyclopedia of Genes and Genomes (GO/KEGG) pathway enrichment analyses were conducted using fold-change–weighted statistics to prioritize coordinated biological programs rather than individual genes [39]. Cellular composition was estimated using MCP-counter [17], a marker-based method designed to infer relative immune and stromal population abundance from bulk or spatial transcriptomic data. Intercellular communication networks were modeled using CellChat [40], which infers ligand–receptor interactions based on known signaling databases and was applied to explore compartment-level communication patterns rather than test differential expression.

### 4.7. Multiplex Immunofluorescence Validation

Dual-color multiplex immunofluorescence (mIF; 2-plex) was performed on single FFPE sections to quantify neutrophils (MPO^+^) and myofibroblasts (α-SMA^+^) in an independent validation cohort (n = 8) (Figure 7B). Images were acquired using an Olympus VS200 digital slide scanner (Tokyo, Japan). Ten ROIs (500 μm diameter) per case were annotated by a pathologist and quantified using Element software (version 6.10.01) [41]. MPO^+^ cells were quantified as cells/μm^2^, and α-SMA^+^ area was calculated as stromal area fraction.

### 4.8. TCGA-PDAC Prognostic Analysis

Normalized bulk transcriptomic and clinical data from TCGA-PDAC (n = 178) were analyzed for exploratory prognostic validation [42]. Patients were stratified into “Prehabilitation-TME index high” (neutrophil-high/fibroblast-low) and other groups using MPO:FAP expression ratios. Overall survival was compared using Kaplan–Meier analysis with log-rank testing.

## 5. Conclusions

Multimodal prehabilitation was associated with transcriptional and protein-level features indicative of a more immune-active and less desmoplastic tumor microenvironment in PDAC. The observed increases in neutrophil-related inflammatory signatures, reductions in fibroblast-associated pathways, and lower activity of several oncogenic signaling programs together point toward a microenvironment that may be more permissive to immune engagement.

The identification of a neutrophil-high/fibroblast-low signature showing an improved survival in TCGA provides preliminary support for the potential clinical relevance of these findings, although larger studies will be required to validate this association.

Taken together, this exploratory work suggests that prehabilitation may have biological effects extending beyond its established benefits for functional readiness, highlighting a possible role for host-level interventions in shaping the PDAC tumor microenvironment. Future prospective studies integrating biological, functional, and clinical endpoints will be important for determining whether these microenvironmental changes contribute to improved oncologic outcomes.

## Figures and Tables

**Table 1:** Clinical Characteristics of Prehabilitation and Control Patient Cohorts.

Characteristic	Prehabilitation (n = 6)	Control (n = 6)
**Demographics**		
Age, years (mean ± SD)	70 ± 5	70 ± 12
Sex, n (%)	83% F, 17% M	60% F, 40% M
BMI, kg/m^2^ (mean ± SD)	25.8 ± 8.9	29.2 ± 8.7
Unintentional weight loss in prior 6 months, (mean ± SD)	9.4% ± 10%	10% ± 4.6%
Serum albumin, g/dL (mean ± SD)	3.5 ± 0.4	3.3 ± 0.2
Oral nutrition supplement n (%)	100% Y 0% N	0% Y 100% N
Outpatient RDN assessment	100% Y 0% N	0% Y 100% N
**Comorbidities**		
Hypertension, n (%)	50%	100%
Type 2 diabetes mellitus, n (%)	50%	20%
Coronary artery disease, n (%)	33%	0%
Chronic obstructive pulmonary disease, n (%)	0%	0%
Chronic kidney disease, n (%)	0%	0%
**Tumor and treatment characteristics**		
Surgery type (Whipple/Distal), n (%)	Whipple 67% Distal 33.3%	Whipple 80% Distal 20%
Tumor size, (mean ± SD)	2.7 ± 1.3	3.2 ± 0.2
Receipt of neoadjuvant chemotherapy, n (%)	60% Y 40% N	20% Y 80% N
Receipt of neoadjuvant radiation, n (%)	17% Y 83% N	0% Y 100% N
**Pathology variables**		
Tumor grade (well/moderate/poor), n (%)	Well 16.7% Moderate 33.3% Poor 50%	Well 20% Moderate 60% Poor 20%
Positive lymph nodes (mean ± SD)	0.7 ± 1.2	3.8 ± 2.4
Total lymph nodes removed (mean ± SD)	17.8 ± 4.8	18.2 ± 4.4
**Postoperative complications**		
Any complication ≥ Grade II, n (%)	33.3% Y 66.7% N	60% Y 40% N
Wound infection, n (%)	16.7% Y 83.3% N	20% Y 80% N

## Data Availability

All data required to evaluate the conclusions of this study are included within the article. The raw GeoMX digital spatial profiling data from patient tumor samples have been deposited in the Gene Expression Omnibus (GEO) under accession number GSE313657.

## Supplementary Materials

The following supporting information can be downloaded at: https://www.mdpi.com/article/10.3390/ijms27020943/s1.

## Abbreviations

α-SMA	Alpha–smooth muscle actin
CAF	Cancer-associated fibroblast
DSP	Digital spatial profiling
ECM	Extracellular matrix
FAP	Fibroblast activation protein
FFPE	Formalin-fixed paraffin-embedded
FDR	False discovery rate
GO	Gene Ontology
GeoMx	GeoMx Digital Spatial Profiling
IFN	Interferon
KEGG	Kyoto Encyclopedia of Genes and Genomes
MAPK	Mitogen-activated protein kinase
mIF	Multiplex immunofluorescence
MCP-counter	Microenvironment Cell Populations counter
MHC	Major histocompatibility complex
MIF	Macrophage migration inhibitory factor
MPO	Myeloperoxidase
NES	Normalized enrichment score
NK cell	Natural killer cell
PanCK	Pan-cytokeratin
PDAC	Pancreatic ductal adenocarcinoma
PD-L1	Programmed death-ligand 1
PI3K/AKT	Phosphoinositide 3-kinase/AKT signaling pathway
ROI	Region of interest
TCGA	The Cancer Genome Atlas
TGF-β	Transforming growth factor beta
TME	Tumor microenvironment
Wnt	Wingless-related integration site signaling pathway
