# Supplementary material for: Prehabilitation as a Biologically Active Intervention Is Associated with the Remodeling of the Pancreatic Tumor-Immune Microenvironment
Source: Int J Mol Sci. 2026 Jan 18;27(2):943. doi: 10.3390/ijms27020943 (PMC12841739; doi:10.3390/ijms27020943)
Supplement: Supplementary file 1 [file ijms-27-00943-s001.zip › ijms-4076834-supplementary.pdf]

Figure S1

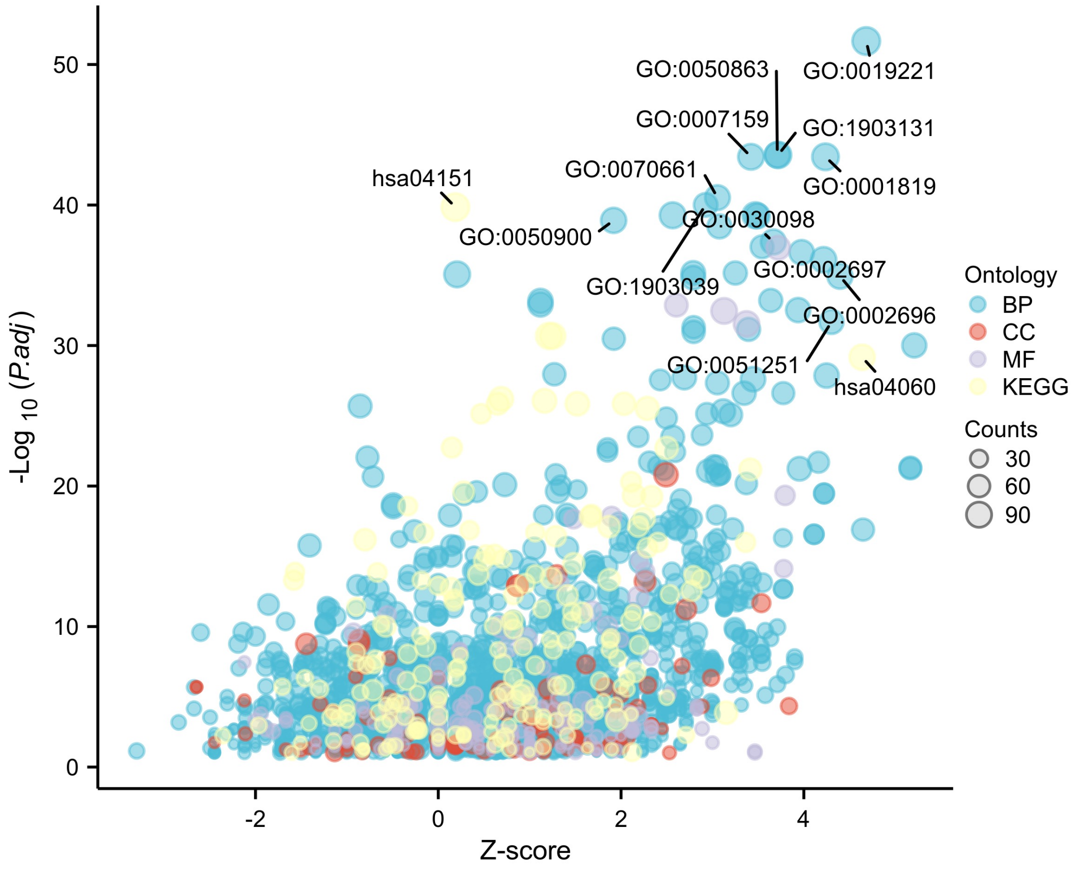

GO:0019221	cytokine-mediated signaling pathway
hsa04151	PI3K-Akt signaling pathway
GO:0001819	positive regulation of cytokine production
GO:1903131	mononuclear cell differentiation
GO:0007159	leukocyte cell-cell adhesion
GO:0002697	regulation of immune effector process
GO:0051251	positive regulation of lymphocyte activation
GO:1903039	positive regulation of leukocyte cell-cell adhesion
hsa04060	Cytokine-cytokine receptor interaction
GO:0050863	regulation of T cell activation
GO:0050900	leukocyte migration
GO:0002696	positive regulation of leukocyte activation
GO:0030098	lymphocyte differentiation
GO:0070661	leukocyte proliferation

Supplementary Figure S1. Bubble plots showing GO/KEGG combined with fold-change pathway enrichment analysis of the differentially expressed genes comparing CD45 regions from prehabilitation-treated and control tumors.

Figure S2

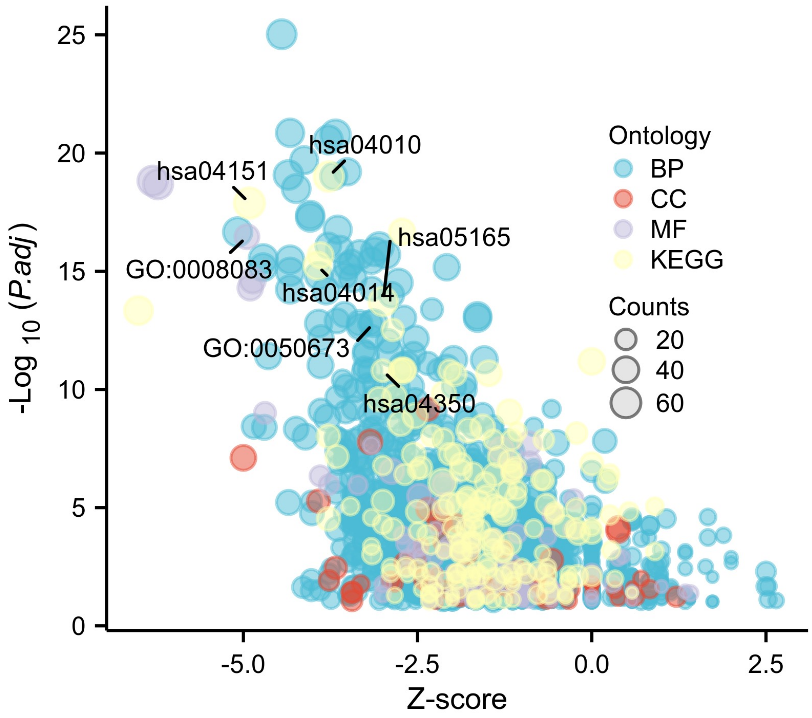

hsa04010	MAPK signaling pathway
hsa04151	PI3K-Akt signaling pathway
GO:0008083	growth factor activity
hsa04014	Ras signaling pathway
hsa05165	Human papillomavirus infection
GO:0050673	epithelial cell proliferation
hsa04350	TGF-beta signaling pathway

Supplementary Figure S2. Bubble plots showing GO/KEGG combined with fold-change pathway enrichment analysis of the differentially expressed genes comparing PanCK+ regions from prehabilitation-treated and control tumors.

Figure S3

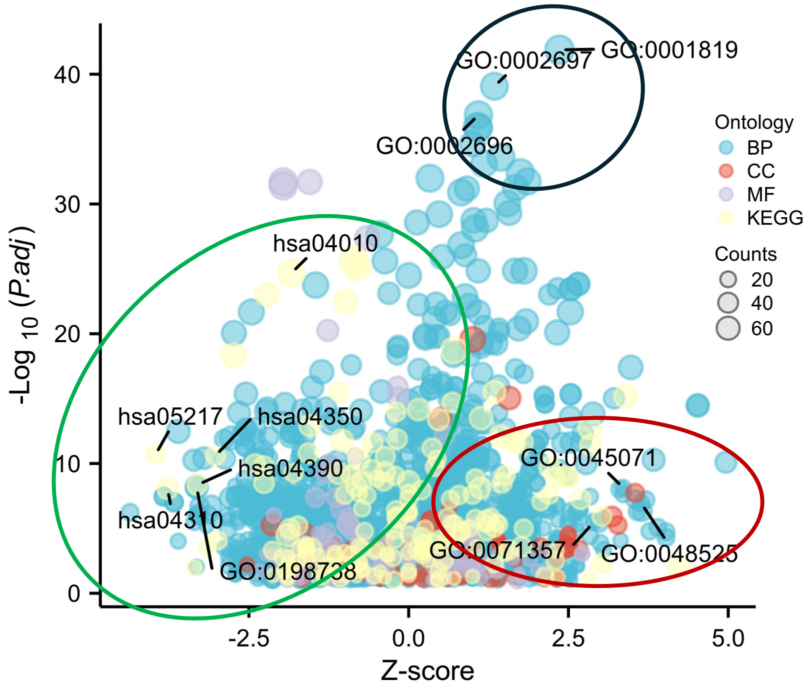

hsa05217	Basal cell carcinoma
hsa04310	Wnt signaling pathway
GO:0198738	cell-cell signaling by wnt
hsa04390	Hippo signaling pathway
hsa04350	TGF-beta signaling pathway
hsa04010	MAPK signaling pathway

GO:0001819	positive regulation of cytokine production
GO:0002697	regulation of immune effector process
GO:0002696	positive regulation of leukocyte activation

GO:0045071	negative regulation of viral genome replication
GO:0071357	cellular response to type I interferon
GO:0048525	negative regulation of viral process

Supplementary Figure S3. Bubble plots showing decreased TGF-β, Wnt, and Hippo signaling (in green circular, negative z scores) and activation of immune-supportive functions (in black circular, positive z scores), including cytokine production, leukocyte activation, and immune effector responses, and negative regulation of viral replication signaling (in red circular, positive z scores) of the differentially expressed genes comparing PanCK-/CD45- stromal regions from prehabilitation-treated and control tumors.
